# A tissue time machine to forward-predict single-timepoint data applied to tuberculosis granulomas

**DOI:** 10.64898/2026.10.09.758083

**Published:** 2026-10-12

**Authors:** Pariksheet Nanda, Erin F. McCaffrey, Christian T. Michael, Alea C. Delmastro, Gabrielle Michael, Joshua T. Mattila, JoAnne L. Flynn, R. Michael Angelo, Denise Kirschner

## Abstract

Recent advances have improved understanding of infection; however many detailed datasets are based on single timepoints post-infection and cannot generate longitudinal insights needed to predict infection outcomes. For example, pulmonary infection with *Mycobacterium tuberculosis* (Mtb) initiates a complex system of immune and bacterial interactions that leads to granuloma formation, yet single snapshots in time need elaboration long-term. We develop a new approach, the *tissue time machine* , applied to predict outcomes of non-human primate granulomas from initiation to endpoint using single timepoint tissue datasets including high-dimensional spatial features. This involves identifying granuloma *digital partners* , simulations most closely resembling real granulomas from a virtual library of over 50,000. By analyzing digital partners, we predict infection outcomes of 22/28 granulomas, and that Mtb-specific, IFN-γ-producing T cell recruitment rates influence uncertainty for the 6/28. This method is applicable to any system that allows for mechanistic predictions over time using heterogeneous, incomplete, and high-dimensional datasets.

